# The treatment of endoscopic third ventriculostomy for hydrocephalus caused by tectal plate gliomas

**DOI:** 10.1186/2045-8118-12-S1-P58

**Published:** 2015-09-18

**Authors:** Jiaping Zheng, Guoqiang Chen, Qing Xiao

**Affiliations:** 1 Aviation General Hospital of China medical university, China, People's Republic of

## Objective

Because of the characteristic of the anatomical location, it is easy for the tectal plate gliomas to cause obstructive hydrocephalus. Endoscopic third ventriculostomy is used for the treatment of obstructive hydrocephalus caused by tectal plate gliomas of the children. Evaluate the prognosis of patients with postoperative follow-up.

## Methods

Preoperative MRI prompted that there were tegmental region masses in 13 children with hydrocephalus. The increase was not obvious. The fiber neuroendoscopy was used for the endoscopic third ventriculostomy. During the operative observation of mesencephalon tegmental region, the hyperplasia of periaqueductal nerve tissue with light pink rough surface and the occlusive aqueduct opening could be observed.

## Results

Followed up for 1-8 years, the average follow-up is 3.6 years. After the operation, 13 patients with the symptoms of headache and unclear vision were in remission and the ventricle was decreased. Postoperative symptom was not in remission in two cases of patients with preoperative diplopia. The treatment of gamma knife was used in one case of patient. The patient was died of radiation encephalopathy after 1 year. Six months after the operation, the symptom of diplopia appeared in 1 case of patient. Reexamination of magnetic resonance imaging (MRI) showed that the tumor increased. 6 months after tumor resection, the patient died. One patient was lost to follow-up. 10 patients survive more than five years.

## Discussion

Incidence of brainstem glioma in children can be accounted for within a 10-20% of primary tumors. The tectal plate gliomas belongs to a rare type. The incidence of glioma in children is less than 5%. Slow growth, years of follow-up imaging results are stable. Most of the patients with glioma of mesencephalon tegmental region are seeing a doctor due to the hydrocephalus. The treatment of endoscopic third ventriculostomy for obstructive hydrocephalus caused by tectal plate gliomas is an effective means. After alleviating hydrocephalus, the patients can live for a long time. Regular follow-up with MRI is needed for the patients.

